# Clinical Characteristics, Management, and Control of Permanent vs. Nonpermanent Atrial Fibrillation: Insights from the RealiseAF Survey

**DOI:** 10.1371/journal.pone.0086443

**Published:** 2014-01-31

**Authors:** Jan Murin, Lisa Naditch-Brûlé, Sandrine Brette, Chern-En Chiang, James O’Neill, P. Gabriel Steg

**Affiliations:** 1 Department of Internal Medicine and Cardiology, Comenius University, Bratislava, Slovakia; 2 Global Strategic Unit Cardio-Thrombosis, Sanofi, Paris, France; 3 Statistician, Lincoln, Boulogne-Billancourt, France; 4 General Clinical Research Center, Taipei Veterans General Hospital and National Yang-Ming University, Taipei, Taiwan; 5 Connolly/Mater Hospitals/Royal College of Surgeons in Ireland, Dublin, Ireland; 6 Institut national de la santé et de la recherche médicale-U698, Paris, France; Université Paris Diderot, Sorbonne Paris Cite, Paris, France; Assistance publique – Hôpitaux de Paris, Hôpital Bichat, Paris, France; University Hospital of Würzburg, Germany

## Abstract

**Background:**

Atrial fibrillation can be categorized into nonpermanent and permanent atrial fibrillation. There is less information on permanent than on nonpermanent atrial fibrillation patients. This analysis aimed to describe the characteristics and current management, including the proportion of patients with successful atrial fibrillation control, of these atrial fibrillation subsets in a large, geographically diverse contemporary sample.

**Methods and Results:**

Data from RealiseAF, an international, observational, cross-sectional survey of 10,491 patients with atrial fibrillation, were used to characterize permanent atrial fibrillation (N = 4869) and nonpermanent atrial fibrillation (N = 5622) patients. Permanent atrial fibrillation patients were older, had a longer time since atrial fibrillation diagnosis, a higher symptom burden, and were more likely to be physically inactive. They also had a higher mean (SD) CHADS_2_ score (2.2 [1.3] vs. 1.7 [1.3], p<0.001), and a higher frequency of CHADS_2_ score ≥2 (67.3% vs. 53.0%, p<0.001) and comorbidities, most notably heart failure. Physicians indicated using a rate-control strategy in 84.2% of permanent atrial fibrillation patients (vs. 27.5% in nonpermanent atrial fibrillation). Only 50.2% (N = 2262/4508) of permanent atrial fibrillation patients were controlled. These patients had a longer time since atrial fibrillation diagnosis, a lower symptom burden, less obesity and physical inactivity, less severe heart failure, and fewer hospitalizations for acute heart failure than uncontrolled permanent atrial fibrillation patients, but with more arrhythmic events. The most frequent causes of hospitalization in the last 12 months were acute heart failure and stroke.

**Conclusion:**

Permanent atrial fibrillation is a high-risk subset of atrial fibrillation, representing half of all atrial fibrillation patients, yet rate control is only achieved in around half. Since control is associated with lower symptom burden and heart failure, adequate rate control is an important target for improving the management of permanent atrial fibrillation patients.

## Introduction

Atrial fibrillation (AF) is associated with substantial morbidity and mortality, as well as having a negative impact on quality of life and exercise capacity compared to the general population [1]. The prevalence of AF is increasing due to longer life expectancy and increased survival rates of patients with cardiovascular (CV) disease [2], [3]. The 2006 ESC guidelines for AF management distinguished three types of AF: paroxysmal AF, persistent AF, and permanent AF (PermAF) [4]. In addition, the first episode of AF has yet to be classified and forms a separate entity. Along with paroxysmal and persistent AF, newly diagnosed AF constitutes nonpermanent AF (nonPermAF). Because the management of AF has historically focused on the restoration and maintenance of sinus rhythm, there is considerably less information regarding PermAF than nonPermAF. The findings from several prospective clinical trials suggest no difference in clinical outcomes when using a rhythm- or rate-control strategy for AF [5]–[8]. At the same time, a retrospective analysis of the AFFIRM trial demonstrated that patients in sinus rhythm at the end of follow-up had improved outcomes compared to patients with AF [9]. Therefore, there is renewed interest in understanding and describing the prevalence, clinical status, and management of patients with PermAF.

Thus far, most of the relevant clinical data available for patients with AF have been limited in that they were derived from single continents (North America or Europe) and often excluded patients with PermAF or had highly selective patient inclusion criteria [2], [10]–[12]. The Real-life global survey evaluating patients with Atrial Fibrillation (RealiseAF) is an international, cross-sectional, observational survey of more than 10,000 patients with AF that aims to describe the AF characteristics, clinical presentation, symptom burden, history of CV events, and comorbidities of AF patients. Importantly, it also aims to describe the management strategies used in real-life practice across the various types of AF, and differences between patients with controlled and uncontrolled AF [13].

The aims of the present analysis of the RealiseAF survey were to: 1) describe in detail the clinical characteristics, risk profile, and management of patients with PermAF compared with nonPermAF; and 2) to characterize those patients with controlled vs. uncontrolled PermAF. These aims were achieved, as described below.

## Methods

### Ethics Statement

The RealiseAF survey was conducted with the approval of the 123 appropriate boards (Table S1) in each of the 26 participating countries. Signed, written informed consent was obtained from all patients or legal representatives. The results of the RealiseAF survey are reported in accordance with the STROBE (STrengthening the Reporting of Observational studies in Epidemiology) statement (www.strobe-statement.org).

### Design

The design, patient population, and data collection process of the RealiseAF survey have previously been described [13]. In summary, RealiseAF included data from patients with current AF or a history of at least 1 AF episode in the previous 12 months. Patients were enrolled in 831 sites in 26 countries spanning 4 continents (Table S2); patient and demographic data collection were carried out at a single visit [13]. In order to achieve unbiased recruitment, participating physicians were randomly selected from lists of cardiologists and internists (hospital or office based) in each country, with a predetermined ratio to reflect national practice. To avoid selection bias, each site was asked to enroll 10–30 consecutive patients with AF over a period of <6 weeks.

Paroxysmal, persistent, and PermAF were defined in accordance with the 2006 American College of Cardiology/American Heart Association/European Society of Cardiology guidelines for AF management, which were those in use at the time of data collection [4]. PermAF was defined as AF “where cardioversion has failed or not been attempted”, as the survey was performed before the updated definition of PermAF was published [14]. However, the survey does include AF control based on management guidelines at the time of the survey, i.e., being either in sinus rhythm or in AF with a heart rate (HR) ≤80 beats per minute (bpm) (as defined in the protocol), and also includes the lenient definition of AF control from the updated European Society of Cardiology guidelines (2010) [14], which was evidenced in the RACE II study [15], i.e., in sinus rhythm or in AF with HR <110 bpm (these data are in the Tables S3, S4, S5, S6). Patients were also characterized according to the European Heart Rhythm Association (EHRA) classification of symptom score [14] and CHADS_2_ score which was available at the time of data collection. The updated CHA_2_DS_2_-VASc score is included in tables S1, S2, S3, S4, S5, S6, S7, S8, in which patients were categorized using lenient AF control.

### Statistical Methods

Determination of sample size has been previously described [13]. Population characteristics were summarized as mean and standard deviation for continuous variables, and as count and percentages for qualitative variables. Descriptive analyses were conducted according to PermAF/nonPermAF status, and within PermAF according to AF “control”.

To identify factors associated with the control of AF in patients with PermAF, a multivariate stepwise logistic regression (with a significance level of 20% for entering and of 5% for retaining the variables in the model) was performed; variables included: age by class, gender, country, obesity (body mass index [BMI] ≥30 kg/m^2^), at least one symptom in the previous 7 days (including the day of the visit), time since AF diagnosis by class, presence of left ventricular hypertrophy, history of heart failure (HF) by New York Heart Association (NYHA) class, history of valvular heart disease, therapeutic strategy prior to visit, use of statins in the previous 7 days, and use of angiotensin-converting enzyme (ACE) inhibitors and/or angiotensin II receptor blockers (ARBs), and/or aldosterone in the previous 7 days. Discrimination between models was assessed using c-statistics and calibrated using Hosmer-Lemeshow χ-square statistics. The odds ratios and associated 95% confidence interval for AF control were determined; the multivariate analysis was adjusted for country. Comparisons between subgroups were made using the χ-square test or Student’s *t*-test, as appropriate. A p-value of 0.05 was retained as significant. Analyses were performed using SAS® statistical software, Version 9.2 (SAS Institute, Cary, NC, USA).

## Results

### PermAF vs. nonPermAF

#### Patient characteristics

Among the 10,491 eligible patients, 46.4% had PermAF, while those with paroxysmal (24.8%) and persistent (22.3%) AF, were equally represented among the remaining patients [13]. A small proportion of patients (6.4%) were recruited at the time of their first AF episode and therefore its type could not be determined. Table 1 shows the characteristics of patients with PermAF (controlled [HR ≤80 bpm] and uncontrolled AF) compared with nonPermAF patients. Compared with patients in the nonPermAF group, patients in the PermAF group were, in general, older (23.9 vs. 32.8% were 75 years or more; p<0.001) and had been diagnosed with AF for a longer time (33.2 vs. 76.5 months; p<0.001).

**Table 1:** Patient characteristics.*

	Types of AF					
		Permanent				
	Nonpermanent	All	Controlled AF	Uncontrolled AF	p-value	p-value
	N = 5622	N = 4869	n = 2262	n = 2246	(controlled AF vs. uncontrolled AF)	(nonpermanent vs. permanent)
Age, years						
Mean (SD)	65.2 (12.3)	68.3 (11.8)	69.8 (11.0)	66.4 (12.4)	<0.001	<0.001
Age, %						
≥75 years	23.9	32.8	37.1	27.5	<0.001	<0.001
Gender/age, %					<0.001	<0.001
Male <75 years	45.4	38.7	37.1	40.3		
Male ≥75 years	11.5	17.1	20.8	12.5		
Female <75 years	30.7	28.5	25.8	32.2		
Female ≥75 years	12.4	15.6	16.3	15.0		
Time since AF diagnosis (months)						
Mean (SD)	33.2 (55.4)	76.5 (79.1)	88.3 (85.8)	66.2 (70.2)	<0.001	<0.001
Time since AF diagnosis, %					<0.001	<0.001
<3 months	33.2	5.8	3.5	7.9		
3–6 months	8.6	3.6	2.4	4.2		
6–12 months	12.2	7.7	6.5	8.3		
>12 months	46.0	82.8	87.6	79.5		
EHRA classification, %					<0.001	<0.001
I	28.9	22.9	27.8	18.9		
II	51.4	52.3	51.2	51.0		
III	18.1	22.4	19.0	27.2		
IV	1.6	2.4	2.0	2.9		
Family history of premature CV disease, %	24.1	21.7	21.3	22.6	0.32	0.007
Current smoker, %	11.3	9.0	7.4	10.5	<0.001	<0.001
Physically inactive, %	57.7	65.1	63.4	67.3	0.006	<0.001
Obese (BMI ≥30 kg/m^2^), %	33.0	32.4	30.8	36.3	<0.001	0.53
Hypertension, %	72.8	71.6	71.5	71.2	0.78	0.17
Diabetes mellitus, %	19.4	23.5	22.8	23.8	0.43	<0.001
Dyslipidemia, %	48.0	44.4	44.9	44.2	0.67	<0.001
Number of risk factors,† %					0.38	0.016
0	1.8	1.2	1.1	1.3		
1	7.2	6.8	6.5	7.1		
2	13.8	12.8	13.5	11.9		
≥3	77.1	79.2	78.9	79.7		
CHADS_2_ score, mean (SD)	1.7 (1.3)	2.2 (1.3)	2.2 (1.3)	2.1 (1.3)	0.030	<0.001
CHADS_2_ score, age <75 years,mean (SD)	1.4 (1.1)	1.8 (1.2)	1.8 (1.2)	1.8 (1.2)	0.78	<0.001
CHADS_2_ score distribution, %					0.12	<0.001
0	15.5	9.0	8.6	9.5		
1	31.5	23.7	23.0	25.0		
≥2	53.0	67.3	68.4	65.5		
CHADS_2_ score distribution, age <75 years, %					0.89	<0.001
0	20.3	13.4	13.6	13.0		
1	38.4	31.5	31.7	31.7		
≥2	41.3	55.1	54.7	55.3		
HR (bpm)						
Mean (SD)	81.8 (25.7)	84.0 (19.7)	69.3 (8.5)	98.8 (16.1)	<0.001	<0.001
HR, %					<0.001	<0.001
<60 bpm	15.7	5.5	10.9	0.0		
60–80 bpm	45.2	44.6	89.1	0.0		
80–110 bpm	23.7	39.0	0.0	78.3		
≥110 bpm	15.4	10.9	0.0	21.7		
QTc – Bazett’s (ms)						
Mean (SD)	439.8 (61.7)	435.4 (58.7)	418.1 (51.1)	452.8 (61.0)	<0.001	<0.001
Patients with LVEF assessed within last 12 months, %	80.9	75.3	74.7	77.8		
If assessed, LVEF in classes, %					0.16	<0.001
<35%	5.3	8.2	7.4	9.0		
35%–40%	3.2	5.5	5.3	5.8		
≥40%	91.5	86.4	87.4	85.1		
Echocardiography performed within last 12 months, %	80.5	75.4	74.9	77.2		
If performed, left atrial size (<50 mm), %	79.6	54.5	50.3	59.4	<0.001	<0.001

AF, atrial fibrillation; BMI, body mass index; BPM, beats per minute; CV, cardiovascular; EHRA, European Heart Rhythm Association; HR, heart rate; LVEF, left ventricular ejection fraction; NA, not available; SD, standard deviation.

CHADS_2_, congestive heart failure, hypertension, age ≥75 years, diabetes, prior stroke or TIA (doubled); CHA_2_DS_2_-VASc, congestive heart failure, hypertension, age ≥75 years (doubled), diabetes, prior stroke or TIA (doubled), vascular disease, age 65–74 years and sex category (female).

* Data are not complete for all patients: the reported percentage is for the number of patients with data available for each given variable.

† CV risk factors used for this calculation included age >50 years for males/>65 years for females, family history of premature CV disease, family history of premature sudden death, current smoker, no physical activity, obesity, arterial hypertension, diabetes mellitus, and dyslipidemia.

Table S3 shows characteristics of permanent AF patients according to revised definition of control used in the RACE II study, i.e., lenient control (in sinus rhythm or in AF with HR <110 bpm) or no control (no sinus rhythm and in AF with HR ≥110 bpm).

PermAF patients had a greater prevalence of AF-related symptoms (EHRA Classes III and IV 22.4% and 2.4%, respectively) compared with nonPermAF patients (18.1% and 1.6%, respectively), and had more CV risk factors than patients with nonPermAF (p = 0.016). Physical inactivity and diabetes mellitus were also more frequent in the PermAF cohort, while family history of premature CV disease, current smoking, and dyslipidemia were more frequent in the nonPermAF cohort. The proportion of patients with CHADS_2_ score ≥2 was higher in patients with PermAF than with nonPermAF (67.3% vs. 53.0%, respectively; p<0.001). Mean (SD) CHADS_2_ scores were also higher in patients with PermAF (2.2 [1.3] vs. 1.7 [1.3], p<0.001) [Table 1]. Mean CHADS_2_ scores were lower in patients aged <75 years than in the overall group, as were the proportions of patients with CHADS_2_ scores ≥2 (Table 1).

Major CV comorbidities were consistently more prevalent in patients with PermAF than with nonPermAF, as summarized in Table 2: patients with PermAF more frequently experienced advanced (NYHA Class III or IV) HF, valvular disease, coronary and cerebrovascular artery disease, and peripheral arterial diseases than nonPermAF patients.

**Table 2:** Comorbidities (%).*

	Types of AF					
		Permanent				
	Nonpermanent	All	Controlled AF	Uncontrolled AF	p-value	p-value
	N = 5622	N = 4869	n = 2262	n = 2246	(controlled AF vs. uncontrolled AF)	(nonpermanent vs. permanent)
At least one comorbidity	71.5	84.8	85.7	83.6	0.057	<0.001
HF, by NYHA class					<0.001	<0.001
No HF or NYHA I	68.4	50.3	53.3	46.6		
HF NYHA II	21.1	29.5	29.2	29.5		
HF NYHA III or IV	10.5	20.2	17.5	24.0		
Valvular heart disease	18.7	35.8	37.3	33.7	0.011	<0.001
Coronary artery disease	30.6	34.3	34.4	34.3	0.93	<0.001
Cerebrovascular disease	11.1	17.6	18.2	16.6	0.17	<0.001
Peripheral arterial disease	3.4	6.0	6.9	5.4	0.042	<0.001

AF, atrial fibrillation; HF, heart failure; NYHA, New York Heart Association.

* Data are not complete for all patients: the reported percentage is for the number of patients with data available for each given variable.

Table S4 shows comorbidities of permanent AF patients according to revised definition of control used in the RACE II study, i.e., lenient control (in sinus rhythm or in AF with heart rate [HR] <110 beats per minute [bpm]) or no control (no sinus rhythm and in AF with HR ≥110 bpm).

#### CV events and interventions in the last 12 months

CV events leading to hospitalization within the previous 12 months are presented in Table 3. A similar proportion of patients with PermAF and nonPermAF had at least one CV event leading to hospitalization within the last 12 months (29.2 vs. 28.3%, respectively). The most frequently reported CV events leading to hospitalization in the PermAF population were acute decompensated HF (13.6%), stroke (7.4%), acute coronary syndrome (7.3%), arrhythmic or (pro)arrhythmic events (5.3%), and transient ischemic attack (3.0%). Acute decompensated HF and stroke were more frequent in PermAF than in nonPermAF patients. In the PermAF group, acute decompensated HF was approximately four times more frequent after the diagnosis of AF (69.8%) than before (17.3%). However, arrhythmic or proarrhythmic events and supraventricular tachycardia or AF flutter were more frequent in patients with nonPermAF than in those with PermAF. Non-central nervous system peripheral embolic events, pulmonary embolism, and major bleeding events were relatively infrequent (<2.0%) in both groups.

**Table 3:** CV events leading to hospitalization and CV interventions in the last 12 months (%).*

	Types of AF					
		Permanent				
	Nonpermanent	All	Controlled AF	Uncontrolled AF	p-value	p-value
	N = 5622	N = 4869	n = 2262	n = 2246	(controlled AF vs. uncontrolled AF)	(nonpermanent vs. permanent)
CV events leading to hospitalization in the last 12 months						
At least one CV event	28.3	29.2	28.7	30.2	0.27	0.27
Stroke	4.9	7.4	7.3	7.4	0.90	<0.001
Transient ischemic attack	2.6	3.0	3.2	2.9	0.50	0.19
Acute coronary syndrome	8.2	7.3	7.3	7.2	0.83	0.069
Arrhythmic or pro-arrhythmic event	10.1	5.3	6.5	3.8	<0.001	<0.001
Supraventricular tachycardia or atrial flutter	6.5	2.0	1.9	2.0	0.72	<0.001
Ventricular tachycardia, torsade de pointes, or ventricular fibrillation	1.3	1.0	1.2	0.9	0.40	0.21
Acute decompensated HF	9.3	13.6	12.7	15.3	0.014	<0.001
Before AF diagnosis	31.5	17.3	16.8	17.6		
After AF diagnosis	53.6	69.8	71.3	68.		
Non-CNS peripheral embolic events	0.6	1.0	1.1	0.9	0.47	0.019
Pulmonary embolism	1.0	1.1	1.2	1.0	0.41	0.50
Major bleeding	1.4	1.9	2.1	1.7	0.34	0.069
CV interventions in the last 12 months						
At least one CV intervention	11.2	13.7	15.4	12.4	0.004	<0.001
PCI	6.7	5.7	6.0	5.4	0.41	0.025
Valvular surgery	2.0	5.7	6.4	5.3	0.11	<0.001
CABG	1.9	2.3	2.6	2.0	0.18	0.12
Cardiac angioplasty	0.3	0.4	0.6	0.2	0.030	0.67
Other CV interventions	1.7	1.7	2.5	1.0	<0.001	0.77

AF, atrial fibrillation; CABG, coronary artery bypass graft; CNS, central nervous system; CV, cardiovascular; HF, heart failure; PCI, percutaneous coronary intervention.

* Data are not complete for all patients: the reported percentage is for the number of patients with data available for each given variable.

Table S5 shows CV events leading to hospitalization and CV interventions in the last 12 months for permanent AF patients according to revised definition of control used in the RACE II study, i.e., lenient control (in sinus rhythm or in AF with heart rate [HR] <110 beats per minute [bpm]) or no control (no sinus rhythm and in AF with HR ≥110 bpm).

At least one CV intervention occurring in the 12 months before the patient’s visit was reported more frequently in the PermAF cohort than in the nonPermAF cohort (13.7 vs. 11.2%; p<0.001 [Table 3]). The most frequent interventions in the PermAF group were percutaneous coronary intervention and valvular surgery (both 5.7%), followed by coronary artery bypass grafting in 2.3% of patients. There were fewer percutaneous coronary interventions but more frequent valvular interventions in patients with PermAF than with nonPermAF.

#### AF management: cardioversions in the last 12 months

Few cardioversions had been attempted in the last 12 months prior to enrollment in the PermAF cohort (6.4%).

#### Management strategy selected

Physicians indicated using a rate-control strategy far more frequently than a rhythm-control strategy in the PermAF group, both before and at the day of the visit (Table 4). In contrast, the nonPermAF group was predominantly managed with a rhythm-control strategy. Among the 9% of PermAF patients managed with rhythm control, one-third was changed to a rate-control strategy at the day of the visit, whereas only 1.4% of rate-control patients were changed to a rhythm-control strategy. By contrast, among the nonPermAF patients managed by a rhythm-control strategy, a relatively small proportion (8.0%) were changed from rhythm control to rate control, while 19.9% of patients managed by a rate-control strategy were changed from rate to rhythm control (Table 4). The type of anti-arrhythmic drugs (AADs) used in PermAF and nonPermAF is shown in Table S7A. Pharmacologic treatment data are also available for PermAF patients according to lenient control (Table S7B).

**Table 4:** Management strategy chosen for AF (%).*

	Types of AF					
		Permanent				
	Nonpermanent	All	Controlled AF	Uncontrolled AF	p-value	p-value
	N = 5622	N = 4869	n = 2262	n = 2246	(controlled AF vs. uncontrolled AF)	(nonpermanent vs. permanent)
Any type of cardioversion†					0.018	<0.001
None	64.2	93.6	94.5	92.2		
1	21.4	3.0	2.9	3.6		
2	7.5	1.5	1.1	1.9		
>2	6.9	1.9	1.6	2.3		
Strategy before the visit					0.027	<0.001
Rhythm control	56.5	9.0	8.0	10.1		
Rate control	27.5	84.2	85.9	83.2		
Both	0.1	0.0	0.0	0.0		
None	15.9	6.8	6.1	6.7		
Strategy at the end of the visit					<0.001	<0.001
Rhythm control	63.1	7.2	6.4	7.9		
Rate control	30.9	88.3	87.7	89.6		
Both	0.2	0.2	0.1	0.3		
None	5.7	4.3	5.8	2.2		
Evolution from rhythm-control strategy					0.015	<0.001
No change	89.4	60.4	64.4	55.6		
Rate (± rhythm)	8.0	35.7	29.4	41.8		
None	2.6	3.9	6.1	2.7		
Evolution from rate-control strategy					0.12	<0.001
No change	78.3	97.9	97.8	98.1		
Rhythm (± rate)	19.9	1.4	1.2	1.6		
None	1.8	0.6	0.8	0.4		

AF, atrial fibrillation.

Rate (± rhythm): rate control with or without rhythm control; rhythm (± rate): rhythm control with or without rate control.

* Data are not complete for all patients: the reported percentage is for the number of patients with data available for each given variable.

† Including pharmacologic cardioversion with AAD therapy and electrical cardioversion; data are not complete for all patients: the reported percentage is for the number of patients with data available for each given variable.

Table S6 shows management strategy for permanent AF patients according to revised definition of control used in the RACE II study, i.e., lenient control (in sinus rhythm or in AF with heart rate [HR] <110 beats per minute [bpm]) or no control (no sinus rhythm and in AF with HR ≥110 bpm).

### Pharmacologic Treatment Prescribed in the Previous 7 Days

In terms of other treatments used in the week before the day of the visit, anticoagulants and treatments related to HF were more frequently used in PermAF compared with nonPermAF patients, whereas statins and antiplatelet agents were prescribed in slightly fewer PermAF than nonPermAF patients. The use of ARBs was similar in PermAF and nonPermAF patients (Table S7A). Pharmacologic treatment use was still generally higher in PermAF patients controlled according to lenient control compared with uncontrolled PermAF patients (Table S7B).

#### Electrocardiographic and echocardiographic data

Table 1 also presents data from the electrocardiogram on the day of the visit and echocardiographic data obtained within the last 12 months. Patients in the PermAF group had a significantly faster mean HR than those in the nonPermAF group; in addition, a greater proportion of patients in the PermAF group had a HR 80–110 bpm than in the nonPermAF group. The corrected QTc interval (Bazett’s formula) [16] was longer in nonPermAF than PermAF patients. Reduced left ventricular ejection fraction and enlarged left atrium were more frequent in the PermAF than in the nonPermAF population (Table 1).

### Controlled vs. Uncontrolled PermAF

Among the 4869 patients with PermAF, 2262 (50.2%) were controlled. Patients in the controlled AF subgroup were older than those in the uncontrolled AF subgroup. Smoking, physical inactivity and obesity (BMI ≥30.0 kg/m^2^) were more frequent in the uncontrolled vs. the controlled PermAF subgroup. Patients with uncontrolled PermAF had more frequent and severe symptoms of HF (as shown by higher NYHA HF classes) than patients in the controlled PermAF subgroup; acute decompensated HF was also more frequent in uncontrolled than in controlled PermAF patients. However, there were more arrhythmic or (pro)arrhythmic events in the controlled than in the uncontrolled PermAF subgroup. There were also significantly more CV interventions in the controlled PermAF subgroup (Table 3). Overall, patients in the controlled PermAF subgroup experienced fewer symptoms (palpitations, dyspnea, fatigue, dizziness, chest pain, syncope) and hospitalizations than the uncontrolled PermAF group (data not shown).

In terms of pharmacologic treatment in the last 7 days, ARBs, statins, and antithrombotics/oral anticoagulants were more frequently prescribed to patients with controlled PermAF, while digoxin was less frequently prescribed (Table S7A and B).

A multivariate logistic regression analysis found that the main predictors of AF control in patients with PermAF were: age ≥75 years, increased time since AF diagnosis, lack of obesity, use of statin treatment, lack of advanced symptoms of HF, presence of valvular heart disease, and the lack of symptoms in the week before the visit (Table S8).

## Discussion

The main findings of this analysis are that PermAF was by far the most frequent subset of AF encountered in routine clinical practice, representing approximately half of all patients with AF. It is associated with a greater duration of AF, more advanced age, and an increased number of comorbidities. Importantly, among patients with PermAF, controlled AF was only achieved in 50.2%, yet was associated with superior functional status and reduced prevalence and severity of HF.

In the RealiseAF survey [13], PermAF was the most common AF subset in routine clinical practice, with a prevalence rate of 46.4%. These findings are consistent with those of previous contemporary studies, i.e., the Euro Heart Survey on AF [10], the German AFNET registry [17], a Spanish cross-sectional study in primary care [18], and a French cross-sectional outpatient registry, where prevalence rates of AF were 29.0%, 32.7%, 45.3%, and 51.8%, respectively [19]. However, there are currently limited data on the characteristics, risk profile, and management of this condition. Thus, the RealiseAF survey provides an opportunity to both examine the prevalence of PermAF among AF patients in a much larger patient population than has previously been studied [13] and to study PermAF patients with controlled and uncontrolled AF more closely.

In line with previous findings [20]–[23], data from RealiseAF have shown that patients with AF are medically complex, with a number of cardiac and non-cardiac comorbidities. Over time, AF typically progresses from paroxysmal, to persistent, and eventually to “end-stage” or PermAF [10]. In this analysis, patients with PermAF were older than those with nonPermAF and had a longer duration of time since AF diagnosis. In addition, approximately one-third of PermAF patients and a quarter of nonPermAF patients were ≥75 years of age. Underlying heart disease was also typically more severe in patients with PermAF; this was further confirmed by the higher prevalence of CV risk factors and the significantly higher proportion of patients with CHADS_2_ score ≥2. A higher CHADS_2_ score also denotes a higher risk for stroke in patients with PermAF; again this was confirmed by the more frequent stroke events experienced by PermAF patients compared with nonPermAF patients over the previous last 12 months.

The main findings from this analysis suggest that, as AF progresses from nonPermAF to PermAF, there is a concomitant increase in the number of associated comorbidities, especially those with a cardiac background. In RealiseAF, major CV comorbidities such as advanced (NYHA Class III or IV) HF, valvular disease, coronary artery disease, and cerebrovascular and peripheral arterial diseases were consistently more prevalent in patients with PermAF than with nonPermAF. PermAF also appeared to have the greatest symptom burden when compared to patients with paroxysmal or persistent AF.

Cardioversion was attempted in fewer than 10% of PermAF patients in RealiseAF; as expected, this group was predominantly managed with a rate-control strategy. In contrast, cardioversion was attempted at least once in one-third of nonPermAF patients, and over half of this group was managed by a rhythm-control strategy. These findings provide a snapshot of current contemporary routine clinical practice.

In terms of pharmacologic treatment, the increased use of ACE inhibitors, diuretics (aldosterone antagonists and other diuretics), and digoxin in PermAF patients is consistent with the observation that these patients were more likely to have underlying heart disease, particularly HF, than nonPermAF patients.

The findings from the RealiseAF survey have shown that patients with controlled AF (being in sinus rhythm or in AF with a HR ≤80 bpm at rest) experience fewer symptoms and hospitalizations, and therefore potentially have an overall better quality of life than those patients with uncontrolled AF. Based on the multivariate analysis, it appears that age (≥75 years), longer duration of AF treatment, less obesity, greater use of statins, absence of HF, and presence of valvular diseases have contributed to greater AF control in these patients. In addition, and similar to the comparison between the overall PermAF and nonPermAF groups, patients with uncontrolled PermAF experienced more symptoms than the controlled PermAF subgroup. In addition, more patients with controlled PermAF than uncontrolled PermAF had experienced at least one CV intervention in the previous 12 months.

The observation that only about half of the patients in the PermAF group achieved AF control emphasizes the need for more effective and earlier initiation of treatments. Maintenance of sinus rhythm with AADs, such as amiodarone, can decrease AF recurrences, relieve symptoms, and improve the patient’s quality of life, but they have been associated with adverse drug reactions – some potentially life-threatening – and also with a decline in treatment compliance [24], [25]. Indeed, the results of the PALLAS trial underscore that not all AADs are safe in PermAF patients [26]. In fact, drugs commonly used in PermAF patients, such as digoxin and amiodarone, have not been subjected to rigorous morbidity-mortality trials in this setting, and the debate continues regarding whether digoxin use may be associated with increased mortality in AF [27], [28]. Furthermore, as shown in the AFFIRM study and other clinical trials, no survival advantage has been demonstrated with a rhythm-control over a rate-control strategy [5]–[8].

Overall, there remains an unmet need for effective rhythm-control treatments with a good safety profile to control AF, minimize symptoms and complications, and potentially delay AF progression to PermAF when used early. Likewise, there is also an unmet need for rate-control treatments for PermAF, which could decrease the incidence of HF, improve symptomatic status, and reduce the incidence of complications.

### Limitations

The RealiseAF survey should be interpreted with caution given its observational and cross-sectional nature. While its geographic span includes a broad mix of low- and middle-income countries, there are no patients from North America. It also lacks data from Central Africa, where patient characteristics and management are likely to be different. Indeed, data from Cameroon do indicate that presentation and outcomes of AF in sub-Saharan Africa are very different from that seen in higher-income countries, due to a higher prevalence of rheumatic valve disease, more prevalent comorbidities, and a lower use of oral anticoagulants [29].

RealiseAF also excluded patients with fatal complications or participants in clinical trials. Such exclusions can only underestimate the clinical impact of AF. In addition, the HR was assessed at rest and not at exercise; therefore, the results regarding “control” of AF should also be interpreted with caution.

Notably, the survey was performed before the 2010 ESC definition of PermAF was available. The 2006 AF guideline definition of PermAF used in this registry relied on physician judgment, recognizing, as stated in the guideline itself, that “permanent AF definition is often arbitrary” [4]. This definition differs from the more recent 2010 AF guideline definition [14], in which PermAF is said to exist “when the presence of the arrhythmia is accepted by the patient (and physician). However, the 2006 criteria remain relevant in the US and also in patients with longstanding PermAF. In the 2006 guidelines, PermAF overlaps with longstanding persistent AF (>1 year) [4]. In the updated European (2010) guidelines, if a rhythm-control strategy is adopted, then the PermAF is redesignated as “longstanding persistent AF” [14].

The 2006 definition of PermAF may also have influenced management change in this survey. Control of PermAF was based on HR ≤80 bpm. Since this survey was conducted, there have been changes in the management approach to PermAF. The RACE II study recently showed that a lenient control (resting HR <110 bpm) was as effective in preventing the primary composite outcome (death from CV causes, hospitalization for HF, stroke, systemic embolism, bleeding, and life-threatening arrhythmic events) as a strict rate-control strategy (resting HR <80 bpm) in 614 patients with PermAF [15]. This lenient control criterion was also applied to the data from this survey (Tables S3, S4, S5, S6), and resulted in 89.2% (n = 4020/4508) having controlled AF. Those patients with uncontrolled AF had a shorter time since AF diagnosis (Table S3). But there was still a higher proportion of patients with HF NYHA III or IV who were uncontrolled (31.7% vs. 19.4%; p<0.001) – this had not been improved by the updated definition (Table S4). With the earlier definition (HR ≤80 bpm), it was 24.0% vs. 17.5%; p<0.001. Acute decompensated HF was also higher in uncontrolled AF, using the lenient definition of control (19.3% vs. 13.3%; p<0.001) (Table S5). This was similar with the earlier definition (15.3% vs. 12.7%; p<0.014). The mainstay of treatment remained rate control in PermAF patients (89.2%), but evolution to rate control was lower with the lenient definition (30.2%) (Table S6) than the earlier definition (41.8%). Essentially, the profile of uncontrolled PermAF patients remained unchanged when the lenient definition of AF control was applied, and shows that these findings are still relevant to updated management guidelines. Although the application of the CHA_2_DS_2_-VASc score did increase the mean (SD) score to 3.2 (1.7) for all patients, and to 2.6 (1.6) for all patients aged <75 years, it was not possible to determine how a shift toward increased severity with the updated CHA_2_DS_2_-VASc affected management of PermAF patients, and this would only be relevant to the updated criteria of AF control. But the CHA_2_DS_2_-VASc score distribution (≥2) between patients with control or no control based on the lenient definition was more marked with CHA_2_DS_2_-VASc (82.5% vs. 74.7%; p<0.001) than CHADS_2_ (67.5% vs. 62.5%; p<0.033).

However, it must be noted that the lenient target used in the RACE II study was only performed in 614 patients, with a small event rate. The updated 2010 guidelines [14] also acknowledge that acute control (HR 80–100 bpm) is beneficial in patients with symptoms or severe hemodynamic stress followed by a long-term rate-control strategy. Similarly, the AFFIRM study recommend a strict resting HR target of 60–80 bpm [9]. Therefore, more research is required in order to identify the optimal HR threshold required to reduce symptoms and adverse outcomes.

Finally, the survey did not include more detailed information on other interventional strategies, such as catheter ablation or early ablation, which is currently being investigated in the Atrial Fibrillation Progression Trial (ATTEST). The ATTEST trial will determine the effect of early radiofrequency ablation compared with standard drug therapy on progression of paroxysmal AF [30]. However, it was not possible to identify patients with early AF (<12 months) in the current survey or examine the profiles in more detail.

## Conclusions

In this survey, PermAF patients comprised the most frequent and severe subset of AF patients in routine clinical practice. Rate control was achieved in around half of all PermAF patients, and patients with uncontrolled PermAF had more frequent and severe HF symptoms, and a greater likelihood of acute decompensated HF than patients with controlled PermAF. These results suggest that an earlier or more effective treatment of AF to prevent PermAF, along with more effective rate control of PermAF, may minimize the symptom burden and risk of complications of AF, and ultimately improve long-term prognosis. Further prospective studies will be needed to test this hypothesis.

## Supporting Information

Table S1 **Independent ethics committees in participating countries.**
(DOC)

Table S2 **Distribution of sites and patients per country, n (%).**
(DOC)

Table S3 **Characteristics of permanent AF patients according to lenient AF control.**
(DOC)

Table S4 **Comorbidities (%) of permanent AF patients according to lenient AF control.**
(DOC)

Table S5 **CV events leading to hospitalization and CV interventions in the last 12 months (%) in permanent AF patients according to lenient AF control.**
(DOC)

Table S6 **Management strategy chosen for permanent AF patients (%) according to lenient AF control.**
(DOC)

Table S7 **Treatments used in the previous 7 days (%) in permanent AF patients according to AF control (in sinus rhythm or in AF with a HR ≤80 bpm).**
(DOC)

Table S8 **Predictors of AF control for permanent AF patients.**
(DOC)

## References

[1] Thrall G, Lane D, Carroll D, Lip GY (2006) Quality of life in patients with atrial fibrillation: a systematic review. Am J Med 119: 448–419. 10.1016/j.amjmed.2005.10.057 16651058

[2] Go AS, Hylek EM, Phillips KA, Chang Y, Henault LE, et al (2001) Prevalence of diagnosed atrial fibrillation in adults: national implications for rhythm management and stroke prevention: the AnTicoagulation and Risk Factors in Atrial Fibrillation (ATRIA) Study. JAMA 285: 2370–2375. 11343485 10.1001/jama.285.18.2370

[3] Stewart S, Hart CL, Hole DJ, McMurray JJ (2001) Population prevalence, incidence, and predictors of atrial fibrillation in the Renfrew/Paisley study. Heart 86: 516–521. 11602543 10.1136/heart.86.5.516 PMC1729985

[4] Fuster V, Ryden LE, Cannom DS, Crijns HJ, Curtis AB, et al (2006) ACC/AHA/ESC 2006 guidelines for the management of patients with atrial fibrillation: full text. Europace 8: 651–745. 16987906 10.1093/europace/eul097

[5] Hohnloser SH, Kuck KH, Lilienthal J (2000) Rhythm or rate control in atrial fibrillation–Pharmacological Intervention in Atrial Fibrillation (PIAF): a randomised trial. Lancet 356: 1789–1794. 11117910 10.1016/s0140-6736(00)03230-x

[6] Roy D, Talajic M, Nattel S, Wyse DG, Dorian P, et al (2008) Rhythm control versus rate control for atrial fibrillation and heart failure. N Engl J Med 358: 2667–2677. 18565859 10.1056/NEJMoa0708789

[7] Van Gelder IC, Hagens VE, Bosker HA, Kingma JH, Kamp O, et al (2002) A comparison of rate control and rhythm control in patients with recurrent persistent atrial fibrillation. N Engl J Med 347: 1834–1840. 12466507 10.1056/NEJMoa021375

[8] Wyse DG, Waldo AL, DiMarco JP, Domanski MJ, Rosenberg Y, et al (2002) A comparison of rate control and rhythm control in patients with atrial fibrillation. N Engl J Med 347: 1825–1833. 12466506 10.1056/NEJMoa021328

[9] Corley SD, Epstein AE, DiMarco JP, Domanski MJ, Geller N, et al (2004) Relationships between sinus rhythm, treatment, and survival in the Atrial Fibrillation Follow-Up Investigation of Rhythm Management (AFFIRM) Study. Circulation 109: 1509–1513. 15007003 10.1161/01.CIR.0000121736.16643.11

[10] Nieuwlaat R, Prins MH, Le Heuzey JY, Vardas PE, Aliot E, et al (2008) Prognosis, disease progression, and treatment of atrial fibrillation patients during 1 year: follow-up of the Euro Heart Survey on atrial fibrillation. Eur Heart J 29: 1181–1189. 18397874 10.1093/eurheartj/ehn139

[11] Nieuwlaat R, Capucci A, Camm AJ, Olsson SB, Andresen D, et al (2005) Atrial fibrillation management: a prospective survey in ESC member countries: the Euro Heart Survey on Atrial Fibrillation. Eur Heart J 26: 2422–2434. 16204266 10.1093/eurheartj/ehi505

[12] Camm AJ, Breithardt G, Crijns H, Dorian P, Kowey P, et al (2011) Real-life observations of clinical outcomes with rhythm- and rate-control therapies for atrial fibrillation RECORDAF (Registry on Cardiac Rhythm Disorders Assessing the Control of Atrial Fibrillation). J Am Coll Cardiol 58: 493–501. 21777747 10.1016/j.jacc.2011.03.034

[13] Steg PG, Alam S, Chiang CE, Gamra H, Goethals M, et al (2012) Symptoms, functional status and quality of life in patients with controlled and uncontrolled atrial fibrillation: data from the RealiseAF cross-sectional international registry. Heart 98: 195–201. 21948959 10.1136/heartjnl-2011-300550

[14] Camm AJ, Kirchhof P, Lip GY, Schotten U, Savelieva I, et al (2010) Guidelines for the management of atrial fibrillation: the Task Force for the Management of Atrial Fibrillation of the European Society of Cardiology (ESC). Eur Heart J 31: 2369–2429. 20802247 10.1093/eurheartj/ehq278

[15] Van Gelder IC, Groenveld HF, Crijns HJ, Tuininga YS, Tijssen JG, et al (2010) Lenient versus strict rate control in patients with atrial fibrillation. N Engl J Med 362: 1363–1373. 20231232 10.1056/NEJMoa1001337

[16] Funck-Brentano C, Jaillon P (1993) Rate-corrected QT interval: techniques and limitations. Am J Cardiol 72: 17B–22B. 10.1016/0002-9149(93)90035-b 8256750

[17] Nabauer M, Gerth A, Limbourg T, Schneider S, Oeff M, et al (2009) The Registry of the German Competence NETwork on Atrial Fibrillation: patient characteristics and initial management. Europace 11: 423–434. 19153087 10.1093/europace/eun369 PMC2659602

[18] Barrios V, Calderon A, Escobar C, de la Figuera M (2012) Patients with atrial fibrillation in a primary care setting: Val-FAAP study. Rev Esp Cardiol (Engl) 65: 47–53. 10.1016/j.recesp.2011.08.008 22054913

[19] Cohen A, Dallongeville J, Durand-Zaleski I, Bouee S, Le Heuzey JY (2010) Characteristics and management of outpatients with history of or current atrial fibrillation: the observational French EPHA study. Arch Cardiovasc Dis 103: 376–387. 20800801 10.1016/j.acvd.2010.06.001

[20] De Ferrari GM, Klersy C, Ferrero P, Fantoni C, Salerno-Uriarte D, et al (2007) Atrial fibrillation in heart failure patients: prevalence in daily practice and effect on the severity of symptoms. Data from the ALPHA study registry. Eur J Heart Fail 9: 502–509. 17174599 10.1016/j.ejheart.2006.10.021

[21] Kerr CR, Humphries KH, Talajic M, Klein GJ, Connolly SJ, et al (2005) Progression to chronic atrial fibrillation after the initial diagnosis of paroxysmal atrial fibrillation: results from the Canadian Registry of Atrial Fibrillation. Am Heart J 149: 489–496. 15864238 10.1016/j.ahj.2004.09.053

[22] Le Heuzey JY, Breithardt G, Camm J, Crijns H, Dorian P, et al (2010) The RecordAF study: design, baseline data, and profile of patients according to chosen treatment strategy for atrial fibrillation. Am J Cardiol 105: 687–693. 20185018 10.1016/j.amjcard.2009.10.012

[23] Tebbe U, Oeckinghaus R, Appel KF, Heuer H, Haake H, et al (2008) AFFECT: a prospective, open-label, multicenter trial to evaluate the feasibility and safety of a short-term treatment with subcutaneous certoparin in patients with persistent non-valvular atrial fibrillation. Clin Res Cardiol 97: 389–396. 18322636 10.1007/s00392-008-0644-y

[24] Kim MH, Smith PJ, Jhaveri M, Lin J, Klingman D (2011) One-year treatment persistence and potential adverse events among patients with atrial fibrillation treated with amiodarone or sotalol: a retrospective claims database analysis. Clin Ther 33: 1668–1681. 22108302 10.1016/j.clinthera.2011.10.005

[25] Camm AJ, Camm CF, Savelieva I (2012) Medical treatment of atrial fibrillation. J Cardiovasc Med (Hagerstown) 13: 97–107. 22193839 10.2459/JCM.0b013e32834f23e1

[26] Connolly SJ, Camm AJ, Halperin JL, Joyner C, Alings M, et al (2011) Dronedarone in high-risk permanent atrial fibrillation. N Engl J Med 365: 2268–2276. 22082198 10.1056/NEJMoa1109867

[27] Whitbeck MG, Charnigo RJ, Khairy P, Ziada K, Bailey AL, et al (2013) Editor’s choice: Increased mortality among patients taking digoxin–analysis from the AFFIRM study. Eur Heart J 34: 1481–1488. 23186806 10.1093/eurheartj/ehs348

[28] Gheorghiade M, Fonarow GC, van Veldhuisen DJ, Cleland JGF, Butler J, et al (2013) Lack of evidence of increased mortality among patients with atrial fibrillation taking digoxin: findings from post hoc propensity-matched analysis of the AFFIRM trial. Eur Heart J 34: 1489–1497. 23592708 10.1093/eurheartj/eht120 PMC3659306

[29] Ntep-Gweth M, Zimmermann M, Meiltz A, Kingue S, Ndobo P, et al (2010) Atrial fibrillation in Africa: clinical characteristics, prognosis, and adherence to guidelines in Cameroon. Europace 12: 482–487. 20179174 10.1093/europace/euq006

[30] Atrial Fibrillation Progression Trial (ATTEST). Available: http://www.clinicaltrials.gov/ct2/show/NCT01570361?term=attest&rank=2. Acessed 2013 October 22.

