# Supplementary material for: Clinical Characteristics, Management, and Control of Permanent vs. Nonpermanent Atrial Fibrillation: Insights from the RealiseAF Survey
Source: PLoS One. 2014 Jan 31;9(1):e86443. doi: 10.1371/journal.pone.0086443 (PMC3908888; doi:10.1371/journal.pone.0086443)
Supplement: Table S1 — Independent ethics committees in participating countries. (DOC) [file pone.0086443.s001.doc]

**Web-only files**

**Table S1.** Independent ethics committees in participating countries.

| **Country** | **Independent Ethics Committee** |
| --- | --- |
| Algeria | Not applicable |
| Azerbaijan | Not applicable |
| Belgium | Medisch-Ethische Commissie |
|  | Heilig-Hartziekenhuis Roeselare-Menen |
|  | Commissie Medische Ethiek UZ Brussel |
|  | Ethisch Comité - UZ Gent |
|  | Comité voor Medische Ethiek - AZ Klina |
|  | Commission d’Ethique Hospitalo-Facultaire Universitaire de Liège |
|  | Centre Hospitalier Universitaire du Sart Tilman, Commission d’Ethique Biomédicale Hospitalo-Facultaire |
|  | Cliniques Universitaires Saint-Luc |
|  | Comité d'Ethique Hôpital Saint-Joseph |
|  | Grand Hôpital de Charleroi - Site St.-Joseph |
|  | Ethische Commissie - Virga Jesse Ziekenhuis |
|  | Comité d'Ethique du GHdC Sites Notre-Dame et Reine Fabiola |
|  | Grand Hôpital de Charleroi (Site Notre-Dame) |
|  | Comité d’Ethique |
|  | C.H. du Bois de l'Abbaye & Hesbaye |
|  | Comité d’Ethique des Hôpitaux Iris Sud |
|  | Ethische Commissie van Sint-Augustinus |
|  | Gasthuis Zusters Antwerpen |
|  | Comité d'Ethique Hospitalière |
|  | C.H.R. de la Citadelle |
|  | Comité d’Ethique du C.H.U. Brugmann |
|  | Comité d'Ethique |
|  | Clinique Saint-Jean |
|  | Ethische Commissie |
|  | AZ Turnhout (Campus Sint-Elisabeth) |
|  | vzw Emmaüs - Ethisch comité |
|  | Comité d'éthique de l'Hôpital La Madeleine |
|  | Comité d’Ethique - CHU Ambroise Paré |
|  | Ethische Commissie |
|  | Algemeen Stedelijk Ziekenhuis Aalst |
|  | Ethisch Comité A.Z. St.-Elisabeth |
|  | Comité d'Ethique Médicale |
| Bulgaria | Not applicable |
| Czech Republic | Not applicable |
| Egypt | Scientific Research and Ethics Committee, Central Administration for Research and Health Development, Ministry of Health |
| Germany | Ethik-Kommission der Ärztekammer Westfalen-Lippe und der Medizinischen Fakultät der Westfälischen Wilhelms-Universität Münster |
| Hungary | Scientific Research Ethics Committee of the Health Care Scientific Council (ETT-TUKEB) |
| India | Institutional Ethics Committee, Care Foundation |
|  | Sterling Hospitals Ethics Committee |
|  | Institutional Ethics Committee, Fortis Hospital |
|  | Medanta Institutional Review Board |
|  | Ethics Committee of The Heart Care Clinic |
|  | Institutional Review Board, Max Devki Devi Heart & Vascular Institute |
|  | Ethical Review Board |
|  | Institutional Ethics Committee, Frontier Lifeline Pvt. Ltd. |
| Ireland | St Vincent’s Healthcare Group Ethics and Medical Research Committee |
|  | Clinical Research Ethics Committee of the Cork Teaching Hospitals |
|  | Research Ethics Committee, Mater Misericordiae University Hospital and Mater Private Hospital |
|  | Beaumont Hospital Ethics Committee |
|  | HSE Midland Area Research Ethics Committee |
|  | HSE South-Eastern Area Research Ethics Committee |
|  | Research Ethics Committee |
|  | UPMC Beacon Hospital Research Ethics Committee |
|  | St Vincent’s Healthcare Group Ethics and Medical Research Committee |
|  | Clinical Research Ethics Committee of the Cork Teaching Hospitals |
| Italy | COMITATO ETICO DELL´AZIENDA OSPEDALIERO-UNIVERSITARIA OSPEDALI RIUNITI UMBERTO I - G.M. LANCISI - G. SALESI DI ANCONA |
|  | COMITATO DI BIOETICA DELLA FONDAZIONE IRCCS POLICLINICO S. MATTEO DI PAVIA |
|  | COMITATO ETICO SPERIMENTAZIONE CLINICA MEDICINALI DELLA AUSL 8 DI AREZZO |
|  | COMITATO ETICO DELLA PROVINCIA DI MODENA |
|  | COMITATO ETICO DELL'AZIENDA OSPEDALIERA S. GIUSEPPE MOSCATI DI AVELLINO |
|  | COMITATO ETICO DELL´AZIENDA OSPEDALIERA S. ANDREA DI ROMA |
|  | COMITATO ETICO PER LE SPERIMENTAZIONI CLINICHE DI MEDICINALI DELL´AZIENDA OSPEDALIERA COMPLESSO OSPEDALIERO S. GIOVANNI - ADDOLORATA DI ROMA |
|  | COMITATO ETICO DELL´IRCCS OSPEDALE CASA SOLLIEVO DELLA SOFFERENZA DI S. GIOVANNI ROTONDO (FG) |
|  | COMITATO ETICO DELL´AZIENDA OSPEDALIERA OSPEDALE DI CIRCOLO E FONDAZIONE MACCHI DI VARESE |
|  | COMITATO ETICO PER LA SPERIMENTAZIONE CLINICA DELLA PROVINCIA DI TREVISO |
|  | COMITATO ETICO DELL´AZIENDA OSPEDALIERA VINCENZO MONALDI DI NAPOLI |
|  | COMITATO ETICO DELLA ASL CE/1 DI CASERTA |
|  | COMITATO ETICO DI AREA VASTA ROMAGNA DI CESENA E ISTITUTO SCIENTIFICO ROMAGNOLO PER LO STUDIO E LA CURA DEI TUMORI DI MELDOLA (FO) |
|  | COMITATO ETICO DELLA ASL 106 DI TERAMO |
|  | COMITATO ETICO PER LA SPERIMENTAZIONE CLINICA DEI MEDICINALI DELLA AUSL 2 DI LUCCA |
|  | COMITATO ETICO UNICO PER LA PROVINCIA DI PARMA |
|  | COMITATO ETICO DELL´AZIENDA OSPEDALIERA DI COSENZA |
|  | COMITATO ETICO DEL COMPRENSORIO SANITARIO DI BOLZANO |
|  | COMITATO ETICO DELL´AZIENDA OSPEDALIERA UNIVERSITARIA MATER DOMINI DI CATANZARO |
|  | COMITATO ETICO DELLA ASL BAT DI ANDRIA (BT) |
|  | COMITATO ETICO DELL´AZIENDA OSPEDALIERA ISTITUTI OSPITALIERI DI CREMONA |
|  | COMITATO ETICO DELL´AZIENDA OSPEDALIERA S. ANNA E S. SEBASTIANO DI CASERTA |
|  | COMITATO ETICO DELLA AUSL LE DI LECCE |
|  | COMITATO ETICO DELLA AUSL DI LATINA |
|  | COMITATO ETICO INDIPENDENTE DELL´AZIENDA OSPEDALIERA UNIVERSITARIA POLICLINICO TOR VERGATA DI ROMA |
|  | COMITATO ETICO DELLA ASL RM/B DI ROMA |
|  | COMITATO ETICO AZIENDALE DELL´AZIENDA OSPEDALIERO-UNIVERSITARIA S. MARIA DELLA MISERICORDIA DI UDINE |
|  | COMITATO ETICO SCIENTIFICO DELL´AZIENDA OSPEDALIERA UNIVERSITARIA POLICLINICO GAETANO MARTINO DI MESSINA |
|  | COMITATO ETICO DELLA AUSL RM/H DI ALBANO LAZIALE |
|  | COMITATO ETICO AZIENDALE DELL´AZIENDA OSPEDALIERO-UNIVERSITARIA S. MARIA DELLA MISERICORDIA DI UDINE |
| Lebanon | Not applicable |
| Lithuania | Lithuanian Bioethics Committee |
| Mexico | Comisión de Investigación, Etica y Bioseguridad (CIEBI) |
| Morocco | Not applicable |
| Portugal | Local Health Ethics Committee - Hospital do Espirito Santo |
|  | Local Health Ethics Committee - Hospital Prof. Doutor Fernando Fonseca |
|  | Local Health Ethics Committee - Centro Hospitalar do Médio Ave |
|  | Local Health Ethics Committee - Centro Hospitalar do Funchal |
| Russia | Independent interdisciplinary Committee on ethical Expertise of clinical Studies |
| Slovakia | Ethics Committee at Diabetol, Ltd |
|  | Central Ethics Committee at National Institute of Cardiovascular Diseases |
| Spain | CAEC Autonómico de Ensayos Clínicos de Andalucía |
|  | CEIC Hospital San Cecilio |
|  | SERGAS Comité Ético de Investigación Clínica de Galicia |
|  | CEIC Hospital 12 de Octubre |
| Sweden | Regionala etikprövningsnämnden i Stockholm |
| Switzerland | Comitato etico cantonale TI |
|  | Kantonale Ethikkommision AG/SO |
|  | Kantonale Ethikkommission TG |
|  | Ethikkommission des Kantons Luzern |
|  | Commission cantonale valaisanne CCVEM |
|  | Ethikkommission Appenzell Ausserrhoden |
|  | Département de la Santé et des Affaires Sociales |
|  | République et Canton du Jura Service Santé |
|  | Service Santé Publique du Canton Fribourg |
|  | Ethikkommission des Kantons St. Gallen |
|  | Ethikkommission beider Basel EBKK |
|  | Kantonale Ethikkommission Bern |
|  | Kantonale Ethikkommission Zürich |
|  | Commission d'éthique pour la recherche clinique en ambulatoire GE |
|  | Commission cantonale d'éthique de la recherche sur l'être humain VD |
| Taiwan | Joint Institutional Review Board |
|  | IRB, Taipei Veterans General Hospital |
|  | IRB, Cheng Hsin General Hospital |
|  | IRB, Shin Kong Wu Ho-Su Memorial Hospital |
|  | IRB, Mackay Memorial Hospital |
|  | IRB, Taipei Medical University - Municipal Wang-Fang Hospital |
|  | IRB, Cathay General Hospital |
|  | IRB, Taipei Medical University Hospital |
|  | IRB, Min-Sheng Hospital |
|  | IRB, Buddhist Tzu Chi General Hospital - Hualien Tzu Chi Medical Center |
|  | IRB, Lanseed Hospital |
|  | RERC, Far Eastern Memorial Hospital |
|  | IRB, Tungs' Taichung MetroHarbor Hospital |
|  | IRB, China Medical University Hospital |
|  | IRB, Show Chwan Memorial Hospital |
|  | IRB, Changhua Christian Hospital |
|  | IRB, Cheng Ching General Hospital |
|  | IRB, Kaohsiung Medical University Chung-Ho Memorial Hospital |
|  | IRB, Chang-Gung Memorial Hospital |
| Tunisia | Fattouma Bourguiba Hospital IEC |
| Turkey | Not applicable |
| Ukraine | Central Ethics Committee Ministry of Health of Ukraine |
| Venezuela | Comité de Ética del Hospital Universitario de Caracas |
