# Supplementary material for: Clinical Characteristics, Management, and Control of Permanent vs. Nonpermanent Atrial Fibrillation: Insights from the RealiseAF Survey
Source: PLoS One. 2014 Jan 31;9(1):e86443. doi: 10.1371/journal.pone.0086443 (PMC3908888; doi:10.1371/journal.pone.0086443)
Supplement: Table S2 — Distribution of sites and patients per country, n (%). (DOC) [file pone.0086443.s002.doc]

**Web-only files**

**Table S2.** Distribution of sites and patients per country, n (%).

|  | **Participating physicians per country** | **Patients eligible for analysis** |
| --- | --- | --- |
| **Country** | **N=831** | **N=10,523** |
| Algeria | 29 (3.5) | 310 (2.9) |
| Azerbaijan | 15 (1.8) | 150 (1.4) |
| Belgium | 27 (3.2) | 306 (2.9) |
| Bulgaria | 30 (3.6) | 450 (4.3) |
| Czech Republic | 26 (3.1) | 280 (2.7) |
| Egypt | 31 (3.7) | 458 (4.4) |
| Germany | 114 (13.7) | 1074 (10.2) |
| Hungary | 46 (5.5) | 506 (4.8) |
| India | 15 (1.8) | 301 (2.9) |
| Ireland | 9 (1.1) | 229 (2.2) |
| Italy | 17 (2.0) | 255 (2.4) |
| Lebanon | 34 (4.1) | 191 (1.8) |
| Lithuania | 32 (3.9) | 452 (4.3) |
| Mexico | 14 (1.7) | 168 (1.6) |
| Morocco | 25 (3.0) | 250 (2.4) |
| Portugal | 12 (1.4) | 165 (1.6) |
| Russia | 42 (5.1) | 750 (7.1) |
| Slovakia | 37 (4.5) | 439 (4.2) |
| Spain | 44 (5.3) | 487 (4.6) |
| Sweden | 13 (1.6) | 264 (2.5) |
| Switzerland | 37 (4.5) | 402 (3.8) |
| Taiwan | 34 (4.1) | 742 (7.1) |
| Tunisia | 24 (2.9) | 471 (4.5) |
| Turkey | 40 (4.8) | 510 (4.8) |
| Ukraine | 70 (8.4) | 700 (6.7) |
| Venezuela | 14 (1.7) | 213 (2.0) |

Reproduced from Steg, et al. (2012) *Heart* 98: 195–201 (Appendix); BMJ Publishing Group Ltd.
