# Supplementary material for: Clinical Characteristics, Management, and Control of Permanent vs. Nonpermanent Atrial Fibrillation: Insights from the RealiseAF Survey
Source: PLoS One. 2014 Jan 31;9(1):e86443. doi: 10.1371/journal.pone.0086443 (PMC3908888; doi:10.1371/journal.pone.0086443)
Supplement: Table S3 — Characteristics of permanent AF patients according to lenient AF control. (DOC) [file pone.0086443.s003.doc]

Table S3. Characteristics of permanent AF patients according to lenient AF control.*

|  | **Permanent AF** | | |
| --- | --- | --- | --- |
|  | **Controlled AF**  **n=4020** | **Uncontrolled AF**  **n=488** | **p-value**  **(controlled AF vs. uncontrolled AF)** |
| Age, years |  |  |  |
| Mean (SD) | 68.6 (11.5) | 63.9 (13.4) | <0.001 |
| Age, % |  |  | <0.001 |
| <75 years | 66.3 | 79.5 |  |
| ≥75 years | 33.7 | 20.5 |  |
| Gender/age, % |  |  | <0.001 |
| Male <75 years | 38.4 | 41.0 |  |
| Male ≥75 years | 17.8 | 7.8 |  |
| Female <75 years | 27.8 | 38.5 |  |
| Female ≥75 years | 16.0 | 12.7 |  |
| Time since AF diagnosis (months)  Mean (SD) | 80.3 (80.7) | 53.0 (60.1) | <0.001 |
| Time since AF diagnosis, % |  |  | <0.0001 |
| <3 months | 4.7 | 14.3 |  |
| 3–6 months | 3.0 | 5.8 |  |
| 6–12 months | 7.4 | 7.7 |  |
| >12 months | 84.9 | 72.2 |  |
| EHRA classification, % |  |  | <0.0001 |
| I | 24.5 | 14.4 |  |
| II | 52.1 | 42.9 |  |
| III | 21.4 | 36.8 |  |
| IV | 2.0 | 6.0 |  |
| Family history of premature CV disease, % | 21.6 | 24.9 | 0.13 |
| Current smoker, % | 8.7 | 11.3 | 0.06 |
| Physically inactive, % | 65.0 | 68.0 | 0.18 |
| Obese (BMI ≥30 kg/m²), % | 33.2 | 36.3 | 0.18 |
| Hypertension, % | 71.8 | 67.8 | 0.07 |
| Diabetes mellitus, % | 22.8 | 27.1 | 0.03 |
| Dyslipidemia, % | 44.9 | 41.1 | 0.13 |
| Number of risk factors,^†^ % |  |  | 0.001 |
| 0 | 1.0 | 2.5 |  |
| 1 | 6.6 | 8.8 |  |
| 2 | 12.9 | 10.9 |  |
| ≥3 | 79.5 | 77.8 |  |
| CHADS_2_ score distribution, % |  |  | 0.033 |
| <2 | 32.5 | 37.5 |  |
| ≥2 | 67.5 | 62.5 |  |
| CHA_2_DS_2_-VASc score distribution, % |  |  | <0.001 |
| 0 | 5.3 | 7.0 |  |
| 1 | 12.2 | 18.3 |  |
| ≥2 | 82.5 | 74.7 |  |

AF, atrial fibrillation; BMI, body mass index; bpm, beats per minute; CV, cardiovascular; EHRA, European Heart Rhythm Association; HR, heart rate; SD, standard deviation.

CHADS_2_, congestive heart failure, hypertension, age ≥ 75 years, diabetes, prior stroke or TIA (doubled); CHA_2_DS_2_-VASc, congestive heart failure, hypertension, age ≥ 75 years (doubled), diabetes, prior stroke or TIA (doubled), vascular disease, age 65–74 years and sex category (female).

*Data are not complete for all patients: the reported percentage is for the number of patients with data available for each given variable.

^†^CV risk factors used for this calculation included age >50 years for males/>65 years for females, family history of premature CV disease, family history of premature sudden death, current smoker, no physical activity, obesity, arterial hypertension, diabetes mellitus, and dyslipidemia.
