# Supplementary material for: Clinical Characteristics, Management, and Control of Permanent vs. Nonpermanent Atrial Fibrillation: Insights from the RealiseAF Survey
Source: PLoS One. 2014 Jan 31;9(1):e86443. doi: 10.1371/journal.pone.0086443 (PMC3908888; doi:10.1371/journal.pone.0086443)
Supplement: Table S4 — Comorbidities (%) of permanent AF patients according to lenient AF control. (DOC) [file pone.0086443.s004.doc]

Table S4. Comorbidities (%) of permanent AF patients according to lenient AF control.*

|  | **Permanent AF** | | |
| --- | --- | --- | --- |
|  | **Controlled AF** | **Uncontrolled AF** | **p-value** |
|  | **n=4020** | **n=488** | **(controlled AF vs. uncontrolled AF)** |
| At least one comorbidity | 84.6 | 85.2 | 0.71 |
| HF, by NYHA class |  |  | <0.001 |
| No HF or NYHA I | 51.2 | 39.6 |  |
| HF NYHA II | 29.4 | 28.8 |  |
| HF NYHA III or IV | 19.4 | 31.7 |  |
| Valvular heart disease | 35.8 | 32.8 | 0.20 |
| Coronary artery disease | 34.2 | 35.5 | 0.59 |
| Cerebrovascular disease | 17.3 | 18.4 | 0.54 |
| Peripheral arterial disease | 6.2 | 5.1 | 0.35 |

AF, atrial fibrillation; bpm, beats per minute; HF, heart failure; HR, heart rate; NYHA, New York Heart Association.

*Data are not complete for all patients: the reported percentage is for the number of patients with data available for each given variable.
