# Supplementary material for: Clinical Characteristics, Management, and Control of Permanent vs. Nonpermanent Atrial Fibrillation: Insights from the RealiseAF Survey
Source: PLoS One. 2014 Jan 31;9(1):e86443. doi: 10.1371/journal.pone.0086443 (PMC3908888; doi:10.1371/journal.pone.0086443)
Supplement: Table S5 — CV events leading to hospitalization and CV interventions in the last 12 months (%) in permanent AF patients according to lenient AF control. (DOC) [file pone.0086443.s005.doc]

Table S5. CV events leading to hospitalization and CV interventions in the last 12 months (%) in permanent AF patients according to lenient AF control.*

|  | **Permanent AF** | | |
| --- | --- | --- | --- |
|  | **Controlled AF** | **Uncontrolled AF** | **p-value** |
|  | **n=4020** | **n=488** | **(controlled AF vs. uncontrolled AF)** |
| CV events leading to hospitalization in the last 12 months | | | |
| At least one CV event | 28.7 | 35.6 | 0.002 |
| Stroke | 7.1 | 9.4 | 0.06 |
| Transient ischemic attack | 3.0 | 3.3 | 0.73 |
| Acute coronary syndrome | 7.2 | 7.8 | 0.61 |
| Arrhythmic or pro-arrhythmic event | 5.3 | 3.7 | 0.13 |
| Supraventricular tachycardia or atrial flutter | 1.9 | 1.8 | 0.88 |
| Ventricular tachycardia, torsade de pointes, or ventricular fibrillation | 1.0 | 1.2 | 0.71 |
| Acute decompensated HF | 13.3 | 19.3 | <0.001 |
| Before AF diagnosis | 17.3 | 17.0 |  |
| After AF diagnosis | 71.1 | 60.6 |  |
| Non-CNS peripheral embolic events | 1.0 | 1.2 | 0.59 |
| Pulmonary embolism | 1.1 | 1.7 | 0.23 |
| Major bleeding | 2.0 | 0.8 | 0.07 |
| CV interventions in the last 12 months | | | |
| At least one CV intervention | 14.3 | 10.2 | 0.014 |
| PCI | 5.8 | 4.7 | 0.33 |
| Valvular surgery | 6.0 | 4.1 | 0.09 |
| CABG | 2.3 | 2.5 | 0.82 |
| Cardiac angioplasty | 0.4 | 0 | 0.15 |
| Other CV interventions | 1.9 | 0.4 | 0.018 |

AF, atrial fibrillation; bpm, beats per minute; CABG, coronary artery bypass graft; CNS, central nervous system; CV, cardiovascular; HF, heart failure; HR, heart rate; PCI, percutaneous coronary intervention.

*Data are not complete for all patients: the reported percentage is for the number of patients with data available for each given variable.
