# Supplementary material for: Clinical Characteristics, Management, and Control of Permanent vs. Nonpermanent Atrial Fibrillation: Insights from the RealiseAF Survey
Source: PLoS One. 2014 Jan 31;9(1):e86443. doi: 10.1371/journal.pone.0086443 (PMC3908888; doi:10.1371/journal.pone.0086443)
Supplement: Table S6 — Management strategy chosen for permanent AF patients (%) according to lenient AF control. (DOC) [file pone.0086443.s006.doc]

**Table S6. Management strategy chosen for permanent AF patients (%) according to lenient AF control.***

|  | **Permanent AF** | | |
| --- | --- | --- | --- |
|  | **Controlled AF** | **Uncontrolled AF** | **p-value** |
|  | **n=4020** | **n=488** | **(controlled AF vs. uncontrolled AF)** |
| Strategy before the visit |  |  | <0.001 |
| Rhythm control | 8.8 | 11.0 |  |
| Rate control | 85.6 | 75.8 |  |
| Both | 0 | 0 |  |
| None | 5.6 | 13.3 |  |
| Strategy at the end of the visit |  |  | <0.001 |
| Rhythm control | 6.6 | 12.2 |  |
| Rate control | 89.2 | 84.3 |  |
| Both | 0.1 | 0.8 |  |
| None | 4.2 | 2.7 |  |
| Evolution from rhythm-control strategy |  |  | 0.35 |
| No change | 58.2 | 67.9 |  |
| Rate (± rhythm) | 37.2 | 30.2 |  |
| None | 4.5 | 1.9 |  |
| Evolution from rate-control strategy |  |  | 0.23 |
| No change | 98.0 | 97.3 |  |
| Rhythm (± rate) | 1.3 | 2.5 |  |
| None | 0.6 | 0.3 |  |

AF, atrial fibrillation; bpm, beats per minute; HR, heart rate.

Rate (± rhythm): rate control with or without rhythm control; rhythm (± rate): rhythm control with or without rate control.

*Data are not complete for all patients: the reported percentage is for the number of patients with data available for each given variable.
