# Supplementary material for: Clinical Characteristics, Management, and Control of Permanent vs. Nonpermanent Atrial Fibrillation: Insights from the RealiseAF Survey
Source: PLoS One. 2014 Jan 31;9(1):e86443. doi: 10.1371/journal.pone.0086443 (PMC3908888; doi:10.1371/journal.pone.0086443)
Supplement: Table S7 — Treatments used in the previous 7 days (%) in permanent AF patients according to AF control (in sinus rhythm or in AF with a HR ≤80 bpm). (DOC) [file pone.0086443.s007.doc]

Table S7A. Treatments used in the previous 7 days (%) in permanent AF patients according to AF control (in sinus rhythm or in AF with a HR ≤80 bpm).*

|  | **Types of AF** | | | | | **p-value** |
| --- | --- | --- | --- | --- | --- | --- |
|  | **Nonpermanent** | **Permanent** | | | |  |
|  |  | **All** | **Controlled AF** | **Uncontrolled AF** | **p-value** |  |
|  | **N=5622** | **N=4869** | **n=2262** | **n=2246** | **(Controlled AF vs. uncontrolled AF)** | **(Nonpermanent vs. permanent)** |
| Use of ≥1 AAD in the previous 7 days | 85.0 | 90.5 | 89.9 | 91.7 | 0.04 | <0.001 |
| Class Ia | 0.7 | 0.1 | <0.1 | 0.2 | 0.45 | <0.001 |
| Class Ic | 10.9 | 1.8 | 1.1 | 2.2 | 0.01 | <0.001 |
| Class II^†^ | 54.0 | 60.0 | 61.4 | 59.8 | 0.27 | <0.001 |
| Class III | 32.8 | 13.7 | 11.7 | 16.0 | <0.001 | <0.001 |
| Class IV^†^ | 14.9 | 18.1 | 17.5 | 17.1 | 0.75 | <0.001 |
| Amiodarone^‡^ | 28.3 | 12.4 | 10.3 | 14.7 | <0.001 | <0.001 |
| Sotalol | 4.0 | 1.0 | 1.0 | 1.0 | 0.98 | <0.001 |
| Digoxin | 16.1 | 45.2 | 44.0 | 47.9 | 0.01 | <0.001 |
| Other treatments used in the previous 7 days | 12.5 | 26.0 | 26.4 | 26.5 | 0.97 | <0.001 |
| Aldosterone antagonist | 40.2 | 46.3 | 47.4 | 48.5 | 0.46 | <0.001 |
| ACE inhibitors | 22.3 | 22.0 | 23.1 | 18.1 | <0.001 | 0.70 |
| Angiotensin II blockers | 33.2 | 30.0 | 33.1 | 28.1 | <0.001 | <0.001 |
| Statins | 81.8 | 92.6 | 94.6 | 91.3 | <0.001 | <0.001 |
| At least one antithrombotic agent | 43.3 | 34.3 | 29.9 | 36.7 | <0.001 | <0.001 |
| At least one antiplatelet agent | 43.2 | 66.6 | 73.2 | 62.8 | <0.001 | <0.001 |
| At least one oral anticoagulant | 38.7 | 61.4 | 64.0 | 61.0 | 0.037 | <0.001 |

AAD, antiarrhythmic drug; ACE, angiotensin-converting enzyme; AF, atrial fibrillation; bpm, beats per minute; HR, heart rate.

*Data are not complete for all patients: the reported percentage is for the number of patients with data available for each given variable.

^†^Includes for AF use.

^‡^Includes first- and second-line use.

Table S7B. Treatments used in the previous 7 days (%) in permanent AF patients according to lenient AF control *

|  | **Permanent AF** | | |
| --- | --- | --- | --- |
|  | **Controlled AF** | **Uncontrolled AF** | **p-value** |
|  | **n=4020** | **n=488** | **(Controlled AF vs. uncontrolled AF)** |
| Other treatments used in the previous 7 days | 26.3 | 27.9 | 0.46 |
| Aldosterone antagonist | 48.1 | 46.3 | 0.45 |
| ACE inhibitors | 21.4 | 14.3 | <0.001 |
| Angiotensin II blockers | 31.2 | 25.8 | 0.015 |
| Statins | 93.9 | 85.5 | <0.001 |
| At least one antithrombotic agent | 32.0 | 43.9 | <0.001 |
| At least one antiplatelet agent | 70.2 | 49.6 | <0.001 |
| At least one oral anticoagulant | 26.3 | 27.9 | 0.46 |

ACE, angiotensin-converting enzyme; AF, atrial fibrillation; bpm, beats per minute; HR, heart rate.

*Data are not complete for all patients: the reported percentage is for the number of patients with data available for each given variable.

^†^Includes for AF use.

^‡^Includes first- and second-line use.
