# Supplementary material for: Clinical Characteristics, Management, and Control of Permanent vs. Nonpermanent Atrial Fibrillation: Insights from the RealiseAF Survey
Source: PLoS One. 2014 Jan 31;9(1):e86443. doi: 10.1371/journal.pone.0086443 (PMC3908888; doi:10.1371/journal.pone.0086443)
Supplement: Table S8 — Predictors of AF control for permanent AF patients. (DOC) [file pone.0086443.s008.doc]

Table S8. Predictors of AF control for permanent AF patients.^*^

| **Variable** | **OR** | **95% CI** | **p-value** |
| --- | --- | --- | --- |
| Age ≥75 years vs. <75 years | 1.30 | [1.13–1.50 ] | <0.001 |
| At least 1 symptom last week including the day of the visit; No vs. Yes | 1.33 | [1.16–1.53 ] | <0.001 |
| Obesity (BMI ≥30 kg/m²); No vs. Yes | 1.25 | [1.09–1.43 ] | 0.002 |
| Time since first AF diagnosis in months |  |  |  |
| 3–6 vs. <3 | 1.38 | [0.88–2.17 ] | 0.17 |
| 6–12 vs. <3 | 1.83 | [1.27–2.64 ] | 0.001 |
| >12 vs. <3 | 2.18 | [1.62–2.92 ] | <0.001 |
| Valvular heart disease; Yes vs. No | 1.36 | [1.18–1.57 ] | <0.001 |
| Heart failure (NYHA class) |  |  |  |
| None/I vs. III/IV | 1.34 | [1.11–1.62 ] | 0.003 |
| II vs. III/IV | 1.22 | [1.02–1.47 ] | 0.033 |
| Statins in the 7 previous days; Yes vs. No | 1.23 | [1.06–1.42 ] | 0.005 |

AF, atrial fibrillation; BMI, body mass index; CI, confidence interval; NYHA, New York Heart Association; OR, odds ratio.

^*^Based on 4240 patients.

Area under the curve: 0.664.

Hosmer-Lemeshow test: 0.63.

Results are adjusted for country.
